# Report on Diseases of Females

**Published:** 1874-01-15

**Authors:** Hiram Nance

**Affiliations:** Kewanee, Ill.


### MEDICAL EXAMINER.

A

*Semi-Monthly Journal of Medical Sciences.*

EDITED BY N. S. DAVIS, M.D., AND F. H. DAVIS, M.D.

No. II. CHICAGO, JANUARY 15, 1874. VOL. XV.

### Original Communications.

### REPORT ON DISEASES OF FEMALES.

BY HIRAM NANCE, M.D., KEWANEE, ILL. READ BEFORE THE MILITARY TRACT MEDICAL SOCIETY AT GALESBURG, ILL., JANUARY 17, 1874.

MR. PRESIDENT AND GENTLEMEN: Being appointed, with my associates, Drs. Patterson, of Galva, and Heller, of Abingdon, to report on "Obstetrics and Diseases of Women and Children," I addressed my coadjutors on the subject, hoping to be able to give you a full report on this important branch of practical medicine. I am sorry to say that Dr. Patterson pleads indisposition from the effects of cold, rheumatism, etc., and is desirous of handing over all the honor, if honor there be, to one less able to do the subject justice. From Dr. Heller I as yet hear nothing, leading me to infer that we may expect a valuable report from him to-day.

You will excuse me, then, in being brief and strictly practical, as I think all our papers should be, as the time of meeting allowed to our Society is so very limited.

*Pelvic Cellulitis.*—What is pelvic cellulitis? The name tells you: "An inflammation of the cellular tissue about the pelvis." It might also with propriety be called perimetritis, or periperitonitis; for all the tissues around and about the pelvis are liable to take on active inflammation when the cellular tissue of the pelvis is involved. I have been led to write on this subject, not from its frequency, but from its variety, and for the reason that the medical student and the practitioner alike are left almost entirely in the dark; for when they turn to the text-books and look over the index for a guide to search for the history and treatment of pelvic cellulitis, they find that the authors on diseases of females have omitted to mention the disease at all until very recently.

Now, can it be that we are treating a disease that has only recently made its appearance? or have our old authors been treating it under other names more euphonious? I think the latter. But I feel confident that this disease, amongst the ordinary physicians who do not give special attention to diseases of females, has been grossly overlooked.

I was called to see a lady, aged about twenty-four years; married; had always been a little irregular, and suffered at each menstrual period with dysmenorrhœa. A few months before my visit she had been delivered of a large dead child. I was informed that her accouchement had been unusually hard, but no malposition. Her recovery was as rapid as usual, or more so, for she was a plump, healthy woman. On examination of the bowels I found exquisite pain in the left iliac region, extending down through the hypogastric region; in the bladder; uterus, etc. In a few days I found enlargement where the pain first commenced, leading me to diagnose inflammation of the ovaria. The tumor — for such it seemed to be — seemed too low down for ovarian; but what else could I call it, as I knew of no disease to compare it to but this? The pulse was full and rapid; pain excessive; tongue white; mouth dry and parched.

My treatment was antiphlogistic: Gave calomel, with comp. powder jalap, followed by sufficient sulphate magnesia to move the bowels once or twice every day; to ease pain, gave sufficient opiates, and had hot epithems of hops, bran-poultices, etc., continually applied. [Tinct. veratrum viride had not made its appearance in those days, or it would have found a place in this case.] Under this treatment my patient recovered so far as to be able to come to my house, a distance of ten miles. The swelling or tumor still remained very prominent; active symptoms had disappeared. I now gave her sol. of iodine and iodide of potass., in the form of Lugol's sol.; painted tinct. iodine over the swelling every day; kept the bowels moderately open, and gave wine and chalybeates to tone the system up.

Not feeling entirely satisfied with my own diagnosis, I proposed counsel, and sent for an old physician, who came to my house to see her. I described the case to the best of my ability to him, and gave my diagnosis as of some ovarian difficulty. He examined her, and when he came to give his opinion he said: "I think this tumor is in the parietes of the abdomen, in the cellular tissue; and I think it will disappear by resolution; or, it may form an abscess, and have to be opened externally." He could not say, "Your patient has pelvic cellulitis," for no such name could be found in the text-books. He advised a continuance of my treatment; and in due time I had the satisfaction of discharging my patient as well. The swelling disappeared, patient became healthy, and, in two or three years, became a mother.

As I remarked before, this case occurred in my early practice, and I had not seen another similar one until the winter of 1872–3. In the meantime I remembered my first case, and tried to post myself in pelvic diseases, and found those cases fully reported by several medical gentlemen in the *Abstract of Medical Sciences*, edited by William Domett Stone, F.R.C.S.

This second case occured in a young lady aged about twenty-four years; primipara; child three weeks old. Found her suffering severely from pain in the left iliac region, and, on examination, a large round swelling, or tumor, presented itself. I was now prepared to diagnose, and did so with some theory and knowledge to guide me. The patient was weak, prostrate, almost ænemic, with profuse night-sweats; pulse full and wavy. I put her on sufficient black-drops to move the bowels every other day; opiates to ease the severe pain; and quinine and tinct. iron as tonics. Under this treatment, in about three weeks the swelling had disappeared, by resolution, and my patient entirely recovered.

My third case was a young unmarried lady, aged about twenty-two years. I was called to see her on the 30th of August, 1873. She had been subject to menstrual irregularities, connected with dysmenorrhœa, for several years, and recently had suffered very much at every menstrual period. I found her very prostrate; countenance looking haggard and sunken; with occasional abdominal colic pains; tongue coated with heavy white fur, and very dry; urine scanty and high-colored, connected with strangury. On inspecting the bowels I found a very perceptible swelling, or tumor, in the pelvic region, laying a little to the right of the median line. Now, the diagnosis must be made, and the question immediately arose in my mind, "Was this a case of acute cystitis, connected with partial retention of urine and enlargement of the bladder? or, was the difficulty connected with the uterus and its appendages?" It is not a very easy matter to diagnose such cases satisfactorily; but I contented myself by giving full doses of morphine every four hours, alternating with f. e. buchu and sweet spirits nitre.

On my next visit I found my patient somewhat relieved; but reaction had come on with such rapidity that the face was red, pulse 120, and mind wandering. The pain was so excruciating that I continued the opiate, alternating it with gtts. iv. f. e. veratrum viride, each every three hours; had epithems of hops applied over the pelvic region, extending nearly all over the bowels; and emptied the bowels by frequent doses of sulphate magesia and large enema.

On my third visit I found my patient but little better; still suffering severely, and almost entire suppression of urine. I considered it necessary to introduce a catheter, and did so. Passed only about an ounce of high-colored urine. This satisfied me there was no retention, and consequently the enlargement did not depend upon this. I now pretty safely could diagnose pelvic cellulitis; and the sequel proved that I was correct.

The treatment that I have given, with very little variation, was continued for twelve days, at which time the pulse became slow; pain partly ceased; countenance became pale; tongue moist, bowels moved more easily; and every symptom indicated a favorable change. I had told her mother the nature of the disease; that it would probably result in abscess, and discharge either in the vagina peritoneal cavity, or form adhesion with the rectum and discharge per rectum.

On visiting my patient after this favorable change, I found the abscess had discharged large quantities of pus through the rectum, to her great relief; and my anxiety was allayed, for I felt that recovery was certain. Convalescence was soon established, and by the aid of wine, quinine, and iron, my patient soon recovered.

Thus you see that pelvic cellulitis is not entirely confined to married ladies. But where the disease does occur, I think you will always find either a puerperal condition or some menstrual irregularity.

Dr. Brown, of New York, says: "The causes of this disease are chiefly parturition, or abortion, endometritis, ovarian operations, or injuries, exposure to cold, and the escape of fluid into the peritoneum."

Another disease, closely allied to the one under consideration, is *metalithmenia*. By this term I mean an escape of blood, or menstrual secretion, usually occuring at the menstrual period, into the peritoneal cavity. It is also called retrouterine hæmaturia, periuterine hæmaturia, and uterine hæmatocele. The blood or secretion thus escaping may find its way through the cellular tissue around the peritoneum, between the vagina and rectum, between the peritoneum and broad ligaments, and various other connecting tissues, forming a hæmatocele, or abscess, filled with blood, or blood and pus This differs but little from cellulitis, and but little violence would be done to our nomenclature should we classify them together. But where this effusion of blood occurs, it is more likely to terminate in resolution, and more likely to occur in the same person repeatedly. Young ladies suffering from dysmenorrhœa are the patients most liable to its attacks. It evidently depends upon some obstruction to the natural outlet of the sanguineous flow, or an arterial twig has become ruptured within the tissues of the pelvis. Recovery is generally more rapid in this than pelvic cellulitis, and the treatment would vary so little that it is unnecessary to give merely a repetition of what has been said. I will only add that in these diseases the vagina should be kept thoroughly cleansed by very frequent large luke-warm water injections, given through the rubber-valve syringe. It not only cleanses the passage, but it affords much relief by its refrigerating and sedative effects.

Dismissing, for the present, cellulitis, I wish to call your attention to a disease of very frequent occurrence, regarding which but little has been written. I allude to *false conception*. Every woman seems to know that there is such a thing as false conception; and I am frequently interrogated by them, saying, "Doctor, what is 'false conception?'" My reply is, that "It is an effort on the part of an unhealthy ovaria, uterus, or some of the appendages of the generative organs, to form a child, but that the organs are not all in a sufficiently physiological condition to accomplish this desirable end." I ask them if they ever saw a blighted wheat-head, or a fungus in the maize or corn-stalk? and tell them, "Here, in the vegetable kingdom, a blight is produced, and why, in the animal, may not a similar occurrence take place?"

I feel afraid that, where this false conception takes place, the woman is not healthy. I am confident that the genital organs are not; but whether this blight depends entirely upon the woman, or the male semen is partly to blame for it, I will not say; though I believe, in nineteen cases in twenty, the female is at fault.

First symptoms of false conception are not unlike an ordinary conception: menses cease; sometimes morning-sickness, and other symptoms which you are all familiar with. After these have continued for two or three months, the woman does not feel well, and occasionally she will have a dark, coffee-like discharge, or the discharge may be tinged with blood. The patient is usually irritable, weak, and sleeps but little; disturbed by frequent dreams; appetite poor. In this condition we are usually consulted, and, if all the symptoms I have described be present, I usually tell her that I fear she is suffering from false conception, though I guard my diagnosis, and prognosis, by saying that I cannot tell positively until the period of quickening; and, usually, before this time arrives I am sent for in a great hurry, on account of profuse flowing.

On arriving, I usually find her pale, faint, and everything deluged with blood, as we do in ordinary cases of abortion. Pain has usually pretty much ceased; and on vaginal examination I expect to find the false conception clogging up the os uteri, though not enough to prevent continued flowing. Nine times in ten this will be the condition of things. I immediately have the patient placed on her left side, near the edge of the bed, introduce my two fingers, and, if possible, remove the substance. Sometimes I find it very difficult to remove it in this way, and if I can conveniently send for placenta-forceps, do so, and remove it in the same way we would a retained placenta after abortion. If I succeed in getting all the fibrous mass away, I feel quite satisfied that, by good attention and eight or ten days' confinement to bed, my patient will get about well. But if, on examination of the mass, I have reason to believe that some part has been left, I have anxiety, for hæmorrhage may make its appearance eight or ten days later; or, remaining there, it may result in irritative fever, or pyæmia. Removing the mass, I give my patient decoction or f. e. ergot, to secure firm contraction; and in two or three hours, if patient cannot rest, administer an opiate, with strict injunctions to keep in bed at least a week.

In September last I was called to a patient in this condition. When I arrived I found the nurse had raised the head of the patient upon a large pillow. She was as pallid as death; pulse hardly perceptible. I immediately drew the pillow away, and let the head down low; examined, and found everything saturated with blood, and the false conception lodged in the mouth of the womb. On its removal, and the administration of ergot, not an unfavorable symptom succeeded. In a few days I gave wine, iron, etc., and she speedily recovered. On seeing the woman in this condition I was thoroughly aroused to the responsibility of my position, for death seemed to stare me in the face, from the terrible loss of blood; but, by the prompt action, life seemed to be saved.

Another case I will briefly allude to. I was called to see Mrs. T., on the night of November 9th, ult. She had been under my treatment, from July last until within three or four months of this time, for ulceration of the os uteri, connected with endo cervicitis. Her age was about forty years. She had not had a child for thirteen years. The treatment had pretty much restored her health, so much so that I had not seen her to give her any medical attention for the three or four months.

It seems that under the treatment the genital organs had become sufficiently healthy to make an attempt to conceive, and she had believed that she was pregnant, and that now abortion was taking place. As she was suffering from intermittent pains, flowing slightly, and on examination the os uteri was found soft, and a little patulous, I quickly agreed with her, and gave her my opinion that either abortion or false conception would probably be the result. I remained a couple of hours, and as no perceptible change was made in her case, I gave her a good-sized dose of morphine, and left five or six doses more, and advised the nurse to give one every three or four hours, if the pains continued irregularly; but if the pains became quite regular, and much flowing came on, to notify me immediately.

The next night, about the same time, a messenger came in haste. When I arrived found her in great pain, and much frightened from the loss of blood; examined, and found the substance in the mouth of the womb. With some difficulty I removed it, washed it, and inspected it carefully. I did not feel entirely satisfied that it was all away, and again examined her; but, finding nothing in the passage, and the pain and hæmorrhage pretty much ceasing, I left some powders of morphine, with strict injunctions for her to keep her bed for ten or twelve days. I heard nothing from her until the 28th, being eighteen days from the time of the removal of the false conception. I was then summoned again to see her; found her suffering severely with pain in the pubic region, extending to the hypogastric; very little hæmorrhage; mouth of uterus soft and patulous; nothing presenting; pulse nearly natural; surface cool; countenance anxious. On inquiry, found she had been up, and in a wash-room filled with steam, and also had went up stairs in a room where there was no fire. Gave her full opiates, with hop poultices applied over the bowels; jugs of hot water to the feet; temperature of room to be regulated by thermometer, and kept at about seventy or seventy-two degrees. Best California port wine was also administered *ad libitum*. This treatment was continued for three or four days, with very little variation; the bowels still continued tender; no swelling; countenance still anxious; and I could tell, by the expression, that she had pains occasionally. I inquired, every visit, in regard to hæmorrhage; but she persisted there was none to amount to anything; still there was a slight oozing, and it, connected with the soreness, and occasionally a little pain, led me to fear that hæmorrhage might make its appearance at any time, as there was reason to fear a little particle of the membranes of the false conception might have been left. My suspicions proved correct, for I was summoned hurriedly to see her. She was flowing profusely; countenance pale and waxy; pulse slow and weak. Examined and found the vagina full of coagulated blood, and the os completely occluded with the same. On its removal, could not detect anything presenting, but the os was soft and patulous. I still supposed something had been left; but how could I remove it? There was no direct way; and I had to content myself with the administration of ergot and other hæmostatics; gave full doses of f. e. ergot; wine continued; cold vaginal injections; patient to be kept perfectly quiet. Visited her next day; no better; flowing still alarming. Stopped the ergot, and gave acetate of lead every two hours; removed the coagulæ, and with a syringe injected in the womb m. t. iron, one to six parts of water; saturated cloths with same, and used them as a tampon. Third day, no better; removed tampon and again used the iron injection; also gave her cold ice-water injections per anum. On my fourth visit, found the hæmorrhage had ceased, and what discharge there was, was of a dark, offensive character, indicating decomposition of some membranous substance.

Now, I want to call your particular attention, in all cases of false conception, abortion, etc., to be very particular in regard to anything being left in the womb. But let me say that, sometimes, with all our care and knowledge, it cannot be avoided; and then such cases as this I have described are liable to arise; and if our patient escapes with her life we may consider ourselves fortunate. Probably fifty per cent. of such cases will die from hæmorrhage, or its sequels. If death does not directly take place, she becomes irritable, bowels costive, no appetite, loss of sleep, etc., all of which may ultimately result seriously. Where the loss of blood has been great, it seems almost impossible, with all our skill in dietetics, including beef, milk, eggs, rice, and the chalybeates, as medicine, to restore the blood-making process, and save our patient.

In regard to arresting uterine hæmorrhage, much has recently been said about the propriety or impropriety of injecting m. t. of iron, in strength varying from one to ten of water to full strength, in the womb. This is my first experience, and I saw no effect from it. But, was this a test case? I think not; for if a particle of membrane is left, I believe the hæmorrhage will continue until it is away; for the membrane must be attached to the uterine walls, and the arterial twigs will continue bleeding until they can contract; and this contraction cannot take place while the membrane is adhering to them. The only thing that I could really say arrested the hæmorrhage in this case was the ice-water injected per anum; and I would especially urge this treatment upon the Society in similar cases.

The last case reported is still under my care. From the great loss of blood, the patient has been so reduced that she is perfectly ænemic, and is suffering from nausea and occasional vomiting, obstinate constipation, loss of appetite, and occasional abdominal pains. I meet these symptoms as they arise, and am very careful not to administer any harsh or irritating medicine. The treatment principally consists in sub nit. bismuth, grs. v., with calomel, grs. i., every four hours; lime-water and brandy given *ad libitum*; move the bowels by injections, and, to ease the abdominal pain, give opiate and starch injections. As soon as the stomach will bear it, give Nichols' preparation of cinchonia et ferri. Not that I especially prefer this to any other ferruginous preparation, but in cases where the stomach is very irritable, I believe it is the best tonic we can give.

*22d December.*—Patient still growing better; appetite improving; tongue cleaning; countenance pleasant, but pale; can raise up on her elbow in bed, which seems to give her much pleasure, as she has been so very weak; is tired of wine and brandy, so I give Nichols' cinchonia et ferri, sub nit. bismuth, and hard cider; urge her, if the stomach is not sick, to use acids freely, and any article of diet she fancies. I have ceased giving her regular attention, as her improvement is such that I do not think she requires it.

I would next call the attention of the Association, briefly, to the subject of *rigid os uteri in labor*. The State Medical Association met in Rock Island in 1872, and, while in session, this subject was brought up; and I now refer to it in order to denounce the advice there given by some of our most worthy and venerable associates. It was claimed by them that in such cases where the os remained rigid a reasonable length of time, that the physician was justifiable in making cross, or crucial, incisions, nearly through the tissues. Now, in my mind, no more dangerous practice could be advocated; for if you once make the slit, the continued pains will enlarge it, and the rupture will grow larger and larger, until the os is torn clear into the peritoneum. I especially refer you to Ramsbotham's process of parturition for cases where the os has been ruptured by pains alone, without any meddlesome midwifery by the knife. He describes several cases, and in nearly every case death was the result. Now, if death is usually the result of rupture of the os uteri from natural causes, how can we expect to avert it by artificially producing the same thing—and this is what we do—if we divide it during labor? Let me say to you, and especially the younger gentlemen of our Society, that when you have a case of rigid os uteri, notwithstanding the case may be tedious, pains severe, pulse rapid, face flushed, and other unpleasant symptoms, stick to your patient, and give her your undivided attention, connected with such relaxants as will ultimately result favorable; *but do not cut.*

I was extremely sorry to see such doctrines advanced by such worthy men as we find in our State Medical Society; but I was equally happy when, looking over the State transactions for that year, that the Publication Committee had omitted the publication of anything relating to such practice.

In cases of this kind, if we are patient, time will usually accomplish all we desire; but we can do much in hastening the labor by relaxing medicines. Ramsbotham recommends the free use of the lancet; and this practice was general until within a few years. I have used it, I think, very advantageously myself; but since the introduction of veratrum viride and chloroform, I should hesitate to use it until I had used one or both of these remedies. No sensible physician would bleed an

ænemic or scrofulous patient; nor would I bleed a plethoric one until I had tried the effects of veratrum viride, unless there were symptoms of congestion of the brain, or other symptoms of convulsions. In such cases, *bleed*, and bleed freely; and at the same time administer chloroform. Locally soothe the vagina and os uteri with lard, mucilaginous injections, sitting over steam bath, etc. And I am confident that much good is often done, and the labor hastened, by introducing one or two fingers in the os and gradually dilating it. I have found this practice often to be useful when the child's head would be almost resting on the perineum, and the os away up forward or backward. By drawing it gently down, and at same time using the dilating process, much good, I am confident, is the result.

In our recent text-books on the medical and surgical treatment of women, and in nearly every medical journal, you find much is said in regard to ulceration of the os uteri, endo cervicitis, and uterine leucorrhœa. I am glad to be able to briefly bring this subject before you. I think these diseases have not been well understood by the profession until within a few years. The introduction of the duck-bill and quadrivalve speculum has revolutionized the treatment of vaginal and uterine diseases; and the physician who pays especial attention to his female patients suffering from diseases requiring the use of the speculum, will not only satisfy himself that he is curing his patients, but will, in time, receive the thanks and congratulations of his female patrons. Before the introduction of the speculum, it was no uncommon thing to find, in every neighborhood, women confined to bed, partly or all the time, with what they and their physician, too, called "falling of the womb." Now I do not want it understood that I say there is no such disease as prolapsus uteri, but I do say it is a disease of rare occurrence; and those women who have suffered from month to month, and from year to year, with supposed prolapsus uteri, have, in most instances, been suffering from ulceration, or endo-cervicitis; and that, had their medical attendant been sufficiently informed in regard to diseases of females, he could have in most instances cured his patient.

By the use of the improved speculum, and a familiarity with the appearance of the os uteri, the neck of the womb, its peculiar secretions, etc., in the physiological and pathological condition, the physician can readily diagnose his case, and with great certainty found a rational treatment. But I would say that no physician can treat his case successfully without a thorough knowledge of the anatomy of the uterus and its appendages, and their appearance in health and disease. He must know on inspection the character and look of the secretions, whether the amount is largely in excess or not; must know, by the look of the ulcer on the womb, what remedy is most likely to succeed by local application.

I refer to this point especially to draw your attention to a case which recently came under my care, where it was thought advisable to have counsel. The doctor was called (an old practitioner of more than twenty-five years' practice). The case was one of abortion, and this was evidently produced by the diseased condition of the womb, as she had aborted two or three times previously. The patient had been sick two or three weeks with nausea, vomiting, bearing-down pains, etc.; also mucous and pus-like discharges. On vaginal inspection, the womb was found to be partially prolapsed, and the os exquisitively tender to the touch. I made a speculum examination, and found the os terribly ulcerated, and this ulceration extending down the vagina, and as red as a piece of red plush velvet. She was about three months in pregnancy, and nothing but abortion, it seemed, would save her life. Not feeling willing, on my own part, to produce it, was my reason to consent to counsel. I gave my venerable counsel a full history of the case, then introduced the speculum and showed him the condition of the os, vagina, etc. We then introduced Simpson's uterine sound, but no resistance was made, and so I felt convinced that the membranes were ruptured; and, on inquiry of the nurse, found a watery discharge had taken place during the night.

The woman aborted in less than twenty-four hours after our council, and in due time I treated her for ulceration, and in four months she was quite well.

Time will not permit further extension of this report; and I will say, in conclusion, that various remedies are used for local treatment, in these diseases, amongst them nit. silver, tinct. iodine, sul. copper, m. t. iron, caustic potash, acid nitrate of mercury, and fuming nitric acid. While in St. Louis, during the session of the American Medical Association, I attended Dr. Papin's clinique at the Woman's Hospital, and he was using as a local application, to most of his cases, Kennedy's *pinus canadensis*, a dark, tar-like extract, of the consistence of molasses. He claims for it much virtue; and certainly gave it the preference over all other remedies. I have no experience with it, never having used it; but from the doctor's recommendation, would especially call your attention to it. I have had better success, in such cases, with the acid nit. mercury than anything else tried, and have been astonished at its curative effects after applying it to an ulcer, or introducing it in the cervix, up to the fundus of the womb, on a pledget of cotton, on the sound. One writer recommends a piece of cotton saturated with it, and a string attached to it, and introduced into the uterus, and allowed to remain twelve or twenty-four hours. In bad cases of uterine leucorrhœa, I would not hesitate to adopt this practice. I have no fears in regard to its prudent use, nor have I of fuming nitric acid. Such heroic remedies, connected with a well-guarded constitutional treatment, will usually reward us with a cure.

